# Petrophysical assessment of the Hammam Faraun, Matulla and Nubia Reservoirs in the Ashrafi Oil Field, Gulf of Suez

**DOI:** 10.1038/s41598-025-86297-0

**Published:** 2025-01-27

**Authors:** Mohammed Amer, Walid M. Mabrouk, Amr M. Eid, Ahmed Metwally

**Affiliations:** Geophysics Department, Faculty of Science, Cairo University, Giza, 12613 Egypt

**Keywords:** Petrophysical analysis, Hydrocarbon assessment, Lithosaturation-model, Ashrafi oil field, Gulf of Suez, Geophysics, Solid Earth sciences

## Abstract

The Hammam Faraun, Matulla, and Nubia formations in the Ashrafi oil field, in the southern Gulf of Suez, Egypt, are key hydrocarbon reservoirs with significant economic importance. These formations, characterized by their favorable reservoir properties and structural settings, play a crucial role in oil and gas accumulation. Their study provides valuable insights into regional petroleum systems and guides exploration and production activities. The Ashrafi Oil Field is one of the most complex and important areas due to its intricate geological framework, which closely resembles that of the Red Sea. Its proximity to the Red Sea further emphasizes its significance. Therefore, the findings from this study can serve as a valuable analogy for understanding the geology of the Red Sea. This study presents a comprehensive structural interpretation and petrophysical evaluation of the Hammam Faraun, Matulla, and Nubia formations by examining well log data and seismic lines, the research quantifies essential petrophysical parameters that characterize the reservoir properties and hydrocarbon potential of these formations. The Hammam Faraun Member exhibits effective porosity values ranging from 0.15 to 0.25 and water saturation levels between 0.23 and 0.67, indicating a significant capacity for hydrocarbon storage, especially in the northern region where net pay thickness can reach up to 60 ft. The Matulla Formation shows effective porosity values between 0.10 and 0.20, with water saturation levels ranging from 0.31 to 0.41 and net pay thickness varying from 51 to 269 ft, highlighting its substantial hydrocarbon reserves. In contrast, the Nubia Formation, characterized by its uniform sandstone composition, has an effective porosity of approximately 0.18 and a consistent water saturation level of about 0.24, with net pay thicknesses between 72 and 155 ft, marking it as an important target for hydrocarbon exploration. Also, the resulting structural interpretation reveals a series of normal faulted structures, including horsts, half-grabens, and step faults. These faults extend across the area, primarily trending northeast-southwest (clysmic trend), with minor northwest-southeast faults perpendicular to the major faults, creating a complex fault network. Integrating this structures with petrophysical parameters such as shale volume, effective porosity, and hydrocarbon saturation provides critical insights into reservoir quality, informing future exploration and production strategies. The study further underscores the lateral variations in water saturation and net pay thickness across the formations, which are closely linked to facies changes. This thorough analysis enhances our understanding of the geological framework and serves as a vital resource for optimizing hydrocarbon recovery and guiding exploration initiatives in the Ashrafi Oil Field. The findings underline the potential of these formations as significant contributors to the region’s hydrocarbon resources, emphasizing the necessity for ongoing exploration and development efforts. Additionally, the insights gained from this research can facilitate the implementation of advanced recovery techniques, ensuring the efficient utilization of hydrocarbon resources while addressing the challenges associated with reservoir management in the Gulf of Suez region and the Red sea.

## Introduction

The Ashrafi Oil Field, located in the southern Gulf of Suez, Egypt, is a vital site for hydrocarbon exploration and production, characterized by a complex structural and stratigraphic framework shaped by the Gulf of Suez rift system’s tectonic evolution^1–3^. Despite decades of exploration, the field remains a focal point due to its untapped potential and the challenges posed by its geological complexity^4^. As part of one of Egypt’s most prolific petroleum provinces, it hosts reservoirs spanning pre-rift to syn-rift sequences, contributing significantly to the country’s energy reserves. A detailed understanding of its geological framework and the application of effective reservoir selection criteria are essential for optimizing recovery strategies and ensuring efficient resource management. Regionally, the strategic location of the Ashrafi Oil Field near the Red Sea offers valuable insights for exploring similar basins, enhancing the understanding of the Red Sea’s hydrocarbon potential, and supporting economic growth through expanded exploration and production activities.

Formation evaluation is a key component in assessing the viability of potential hydrocarbon reservoirs. In the case of the Ashrafi Oil Field, this process involves interpretation of well logs to assess the physical properties of the subsurface formations. Formation evaluation focuses on determining critical factors such as lithology, porosity, permeability, fluid saturations, and the presence of hydrocarbons. This data is essential in identifying the most promising formations within the field that meet the criteria for economic hydrocarbon production. In this study, a comprehensive formation evaluation approach will be used to highlight the most productive zones within the Ashrafi Oil Field^3,5–10^.

An important aspect of this research is the application of reservoir selection criteria, which helps in determining the best candidates for development and production. In the Ashrafi Oil Field, reservoirs are selected based on factors such as thickness, lateral extent, porosity, permeability, and the presence of structural or stratigraphic traps. Evaluating these criteria is crucial for prioritizing reservoirs that offer the greatest potential for economic hydrocarbon recovery^6–8,11,12^. This study will assess the various reservoir zones in the field, applying selection criteria that focus on maximizing recovery efficiency and minimizing risks associated with production.

The Ashrafi Oil Field exhibits a diverse range of lithologies, with both carbonate and clastic formations playing a major role in its reservoir systems. These rock types have been subject to various diagenetic processes over time such as compaction, cementation, and dissolution which have greatly influenced their reservoir quality^2,3,13,14^. In the carbonates, for example, early cementation has often reduced porosity, while in certain clastic layers, dissolution of minerals has enhanced pore space. However, the overall reservoir characteristics can vary significantly across the field, with some zones benefiting from improved permeability, while others are more complex due to the effects of these diagenetic alterations. Understanding the impact of these processes on the reservoir’s performance is crucial for identifying areas with higher potential for hydrocarbon extraction.

Beyond formation evaluation, the tectonic setting of the Ashrafi Oil Field is crucial in shaping how reservoirs are selected for development. The field lies within the Gulf of Suez rift system, an area marked by intense tectonic activity that has shaped the subsurface landscape over millions of years. This rifting process has generated a complex network of faults, fractures, and structural traps, which serve as natural pathways for hydrocarbon migration and storage^2,3,13,15^. These tectonic features have not only controlled where oil and gas have accumulated but also influenced how easily these resources can be accessed. Some traps may efficiently hold hydrocarbons, while others, due to faulting, may create compartments that limit fluid flow. Understanding this tectonic architecture is essential for pinpointing reservoirs with the best potential for sustainable and profitable extraction.

In summary, this manuscript aims to deliver a detailed formation evaluation and apply reservoir selection criteria for the Ashrafi Oil Field. By integrating geological and petrophysical data, this study provides critical insights into the field’s subsurface properties, enabling the identification and prioritization of the most promising reservoirs. The findings from this research can serve as a valuable analogue for unlocking the hydrocarbon potential of the Red Sea. The insights gained will not only enhance reservoir management strategies and optimize hydrocarbon recovery in the Gulf of Suez but also contribute significantly to future exploration efforts, helping to better understand and harness the Red Sea’s untapped hydrocarbon resources.

### Geological settings

**Fig. 1:**
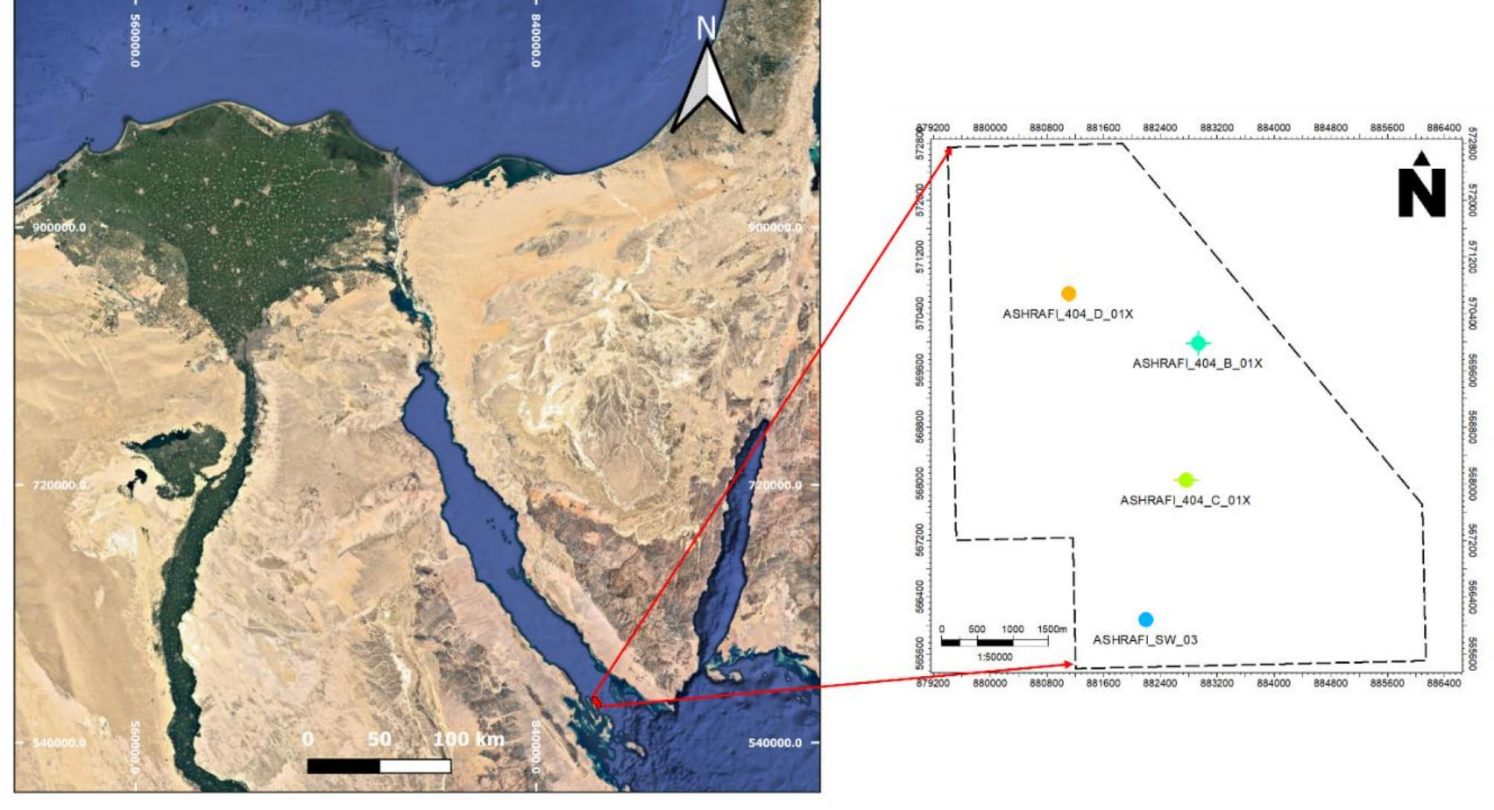
Location map of the study area with a base map showing the four used well locations over Ashrafi oil field.

The Ashrafi Oil Field is located in the southern Gulf of Suez (Fig. 1), a key hydrocarbon-producing region in Egypt. The field lies within the Gulf of Suez rift basin, which has a rich history of tectonic activity that has played a critical role in shaping subsurface geology. The structural and stratigraphic complexities of the area have given rise to a variety of hydrocarbon traps and reservoir types, making it an important target for oil and gas exploration and production. This section provides an overview of the structural framework, stratigraphy, reservoir characteristics, and petroleum system that define the Ashrafi Oil Field^2,3,15,16^.

The Gulf of Suez is a classic example of a rift basin formed during the Oligocene to early Miocene due to the extension and breakup of the African and Arabian plates. The Ashrafi Oil Field lies within the southern part of this rift, where the structural setting is dominated by a series of fault-bounded blocks that dip towards the basin center. These normal faults, which were generated by extensional tectonics, have created tilted fault blocks and horst-and-graben structures, forming numerous structural traps that are conducive to hydrocarbon accumulation^2,3,17,18^.

The field is positioned within a complex system of high-angle faults, with both large-scale faults and smaller secondary fault systems playing an essential role in compartmentalizing the reservoirs. The major faults trend predominantly northwest-southeast, consistent with the overall structural grain of the Gulf of Suez. These faults not only act as trapping mechanisms for hydrocarbons but also influence fluid flow within the reservoirs. In some cases, faulting has created isolated compartments, while in others, it has enhanced permeability by providing pathways for fluid migration.

**Fig. 2:**
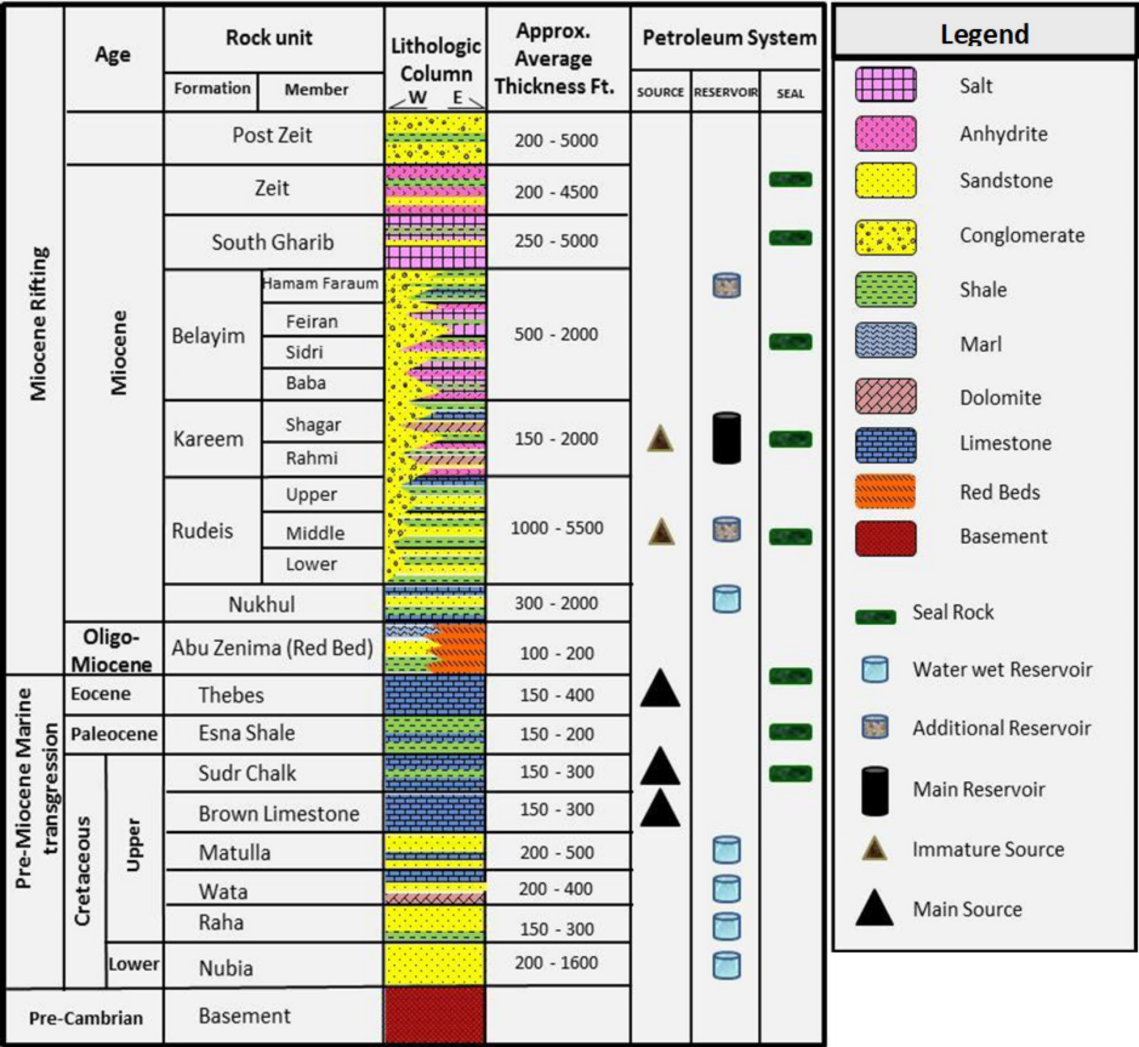
Stratigraphic column of the southern part of the Gulf of Suez^4^.

The sedimentary history of the Gulf of Suez (Fig. 2), spanning from the Late Cretaceous to the Eocene, is marked by significant formations that play critical roles in hydrocarbon exploration and production. The Nubia Formation, characterized by its coarse-grained sandstones, stands out as a primary reservoir due to its advantageous porosity and permeability. Above it, the Matulla Formation introduces lithological variability with its alternating shales and sandstones, influencing hydrocarbon distribution and recovery. The Miocene epoch further contributed to the region’s geological complexity, highlighted by the Nukhul Formation’s marine sediments, which are essential for hydrocarbon generation. The overlying Rudeis Formation provides critical sealing properties with its deep marine shales and marls, preventing hydrocarbon migration. The Kareem Formation, divided into the Markha and Shagar Members, adds further intricacy with its mix of shales, carbonates, and sandstones. Ultimately, the Belayim Formation, encompassing the Hammam Faraun, Feiran, Sidri, and Baba Members, holds significant hydrocarbon potential, with its sandstones and fractured carbonates ensuring excellent reservoir quality, while evaporite deposits play a vital role in hydrocarbon trapping^1–3,18^.

The primary reservoirs of the Ashrafi Oil Field are found in syn-rift sequences, which were deposited during the Oligocene to Miocene rifting events^19^. These sequences consist of both clastic and carbonate formations, with the most significant reservoirs located within the Miocene strata. The Lower Miocene Kareem, Rudeis, and Belayim formations are particularly important, as they contain thick sequences of sandstones and carbonates that serve as the main hydrocarbon-bearing units. The Kareem Formation, for example, is composed of interbedded sandstones, shales, and limestones, providing both reservoir and seal units.

The petroleum system in the Ashrafi Oil Field is closely linked to the tectonic and sedimentary evolution of the Gulf of Suez^20^. The source rocks in this region are primarily organic-rich shales of the Upper Cretaceous and Eocene formations, which were deposited in deep marine environments. These source rocks have undergone significant burial and maturation, generating large volumes of oil and gas over time. The hydrocarbons generated in these source rocks migrated upwards along faults and fractures into the overlying reservoirs, where they became trapped in structural and stratigraphic traps.

The migration pathways in the Ashrafi Oil Field are primarily controlled by the extensional fault systems, which provide vertical and lateral conduits for hydrocarbons to move from the deeper source rocks to shallower reservoir units. Sealing mechanisms in the field are provided by shales and evaporites that act as regional and local seals, preventing the escape of hydrocarbons from the reservoirs^21^.

In terms of fluid composition, the Ashrafi reservoirs primarily contain light to medium-gravity oil, with associated gas present in certain formations. The oil is generally of good quality, with low sulfur content, making it economically attractive for production. However, variations in reservoir pressure and water saturation across the field require careful management to optimize recovery.

The Ashrafi Oil Field, located in the Gulf of Suez, shares geological features with the Red Sea, making it a valuable analogue for exploring hydrocarbon potential in this region. Both areas face challenges such as complex geology, deep waters, and thick salt layers, but the Gulf of Suez has successfully managed these obstacles to unlock significant petroleum resources. Exploration in the Red Sea, though still limited, has revealed a working petroleum system, with increasing interest from countries like Saudi Arabia and Egypt in tapping into its potential​. Therefore, the techniques and findings from the Ashrafi field can provide useful insights for improving exploration and recovery strategies in the Red Sea.

### Data and methodology

Well log data from available four drilled wells named (Ashrafi_404_D_01X, Ashrafi_404_B_01X, Ashrafi_404_C_01X, and Ashrafi_Sw_03) and seismic lines were utilized to conduct a thorough formations detection of the Ashrafi Oil Field. Including a variety of wireline logs, such as Gamma Ray (GR), Density (RHOB), Neutron (NPHI), Sonic (∆T), Photoelectric Factor (PEF), and multiple resistivity measurements (MSFL, LLS, and LLD), facilitated an in-depth identification of the lithologies present in the field.

Each log type provided unique insights into the subsurface geology, enhancing our understanding of the reservoir’s properties. The Density and Neutron logs were especially valuable for assessing porosity and fluid content, while the Gamma Ray log effectively distinguished between clay-rich and cleaner formations. Additionally, the PEF log contributed to a more nuanced interpretation of mineral composition, and the Sonic log supplied essential information regarding the mechanical properties of the formations. The resistivity logs also played a vital role in identifying productive zones and evaluating hydrocarbon saturation. Through the integration of these diverse datasets, we achieved a comprehensive understanding of the reservoir characteristics, which will aid in developing more effective exploration and production strategies in the Ashrafi Oil Field.

The quality control process for wireline logs in the Ashrafi Oil Field involved a meticulous review to ensure the accuracy and reliability of the well log data. Specific procedures, such as error checks for depth mismatches and cross-validation with core data, were applied to identify and correct any inconsistencies or irregularities. This process ensured that the data remained precise and consistent, forming a solid foundation for deriving reliable petrophysical parameters. The petrophysical analysis of key formations, including the Hammam Faraun Member, Matulla, and Nubia Formations, began with a thorough evaluation of the well logs. By rigorously reviewing high-quality data from key wells, the consistency of the logs was confirmed, ensuring that all derived parameters were based on trustworthy measurements. This careful verification process is essential for providing a reliable basis for detailed reservoir characterization and effective decision-making^2^.

Following quality control, the next phase involved the identification of zones of interest within the formations based on the hydrocarbon indicators methods such as Density-Neutron crossover, elevated resistivity values with specific patterns & hydrocarbon saturations from rock models. The PEF log was also instrumental in differentiating between oil and gas, aiding in the assessment of fluid types present within the reservoirs. This was accomplished by analyzing the log signatures to delineate various lithological units and their respective characteristics. The Hammam Faraun Member was highlighted for its significant hydrocarbon potential, characterized by favorable porosity and resistivity values. The Matulla Formation and Nubia Formation were also scrutinized for their reservoir qualities, with careful attention paid to variations in lithology and fluid content that could influence hydrocarbon accumulation^2,6,7,9,10,22,23^.

Matrix identification was a critical step in this analysis, focusing on determining the mineralogical composition of the rock matrix. The integration of Density, Sonic, and PEF logs facilitated the characterization of mineralogy, allowing for accurate calculations of volume fractions of different components, such as quartz, feldspar, and clay. This mineralogical understanding is essential for further quantifying reservoir properties, including porosity and saturation^6,11,23–25^.

The final phase of the petrophysical analysis involved well correlation and mapping of petrophysical parameters across the area based on convergent interpolation. Key parameters such as volume of shale, effective porosity, and water saturation were mapped to visualize their distribution and variations within the Hammam Faraun Member, Matulla Formation, and Nubia Formation. This spatial analysis allowed for the identification of potential high-quality reservoir zones and their respective thicknesses. The mapping results highlighted areas with optimal reservoir characteristics, which are crucial for guiding future exploration and development efforts in the Ashrafi Oil Field.

Following the petrophysical analysis, seismic interpretation was performed across available seismic lines in the depth domain to study the subsurface structural traps. This interpretation aimed to identify key features such as faults, folds, and tectonic elements that influence the petroleum system. By tracking the structural traps and assessing their impact on hydrocarbon accumulation and migration, the seismic analysis provided insights into potential reservoir zones and sealing mechanisms. It also helped identify areas with favorable conditions for hydrocarbon accumulation, guiding future exploration and production strategies.

## Results

### Log display

**Fig. 3:**
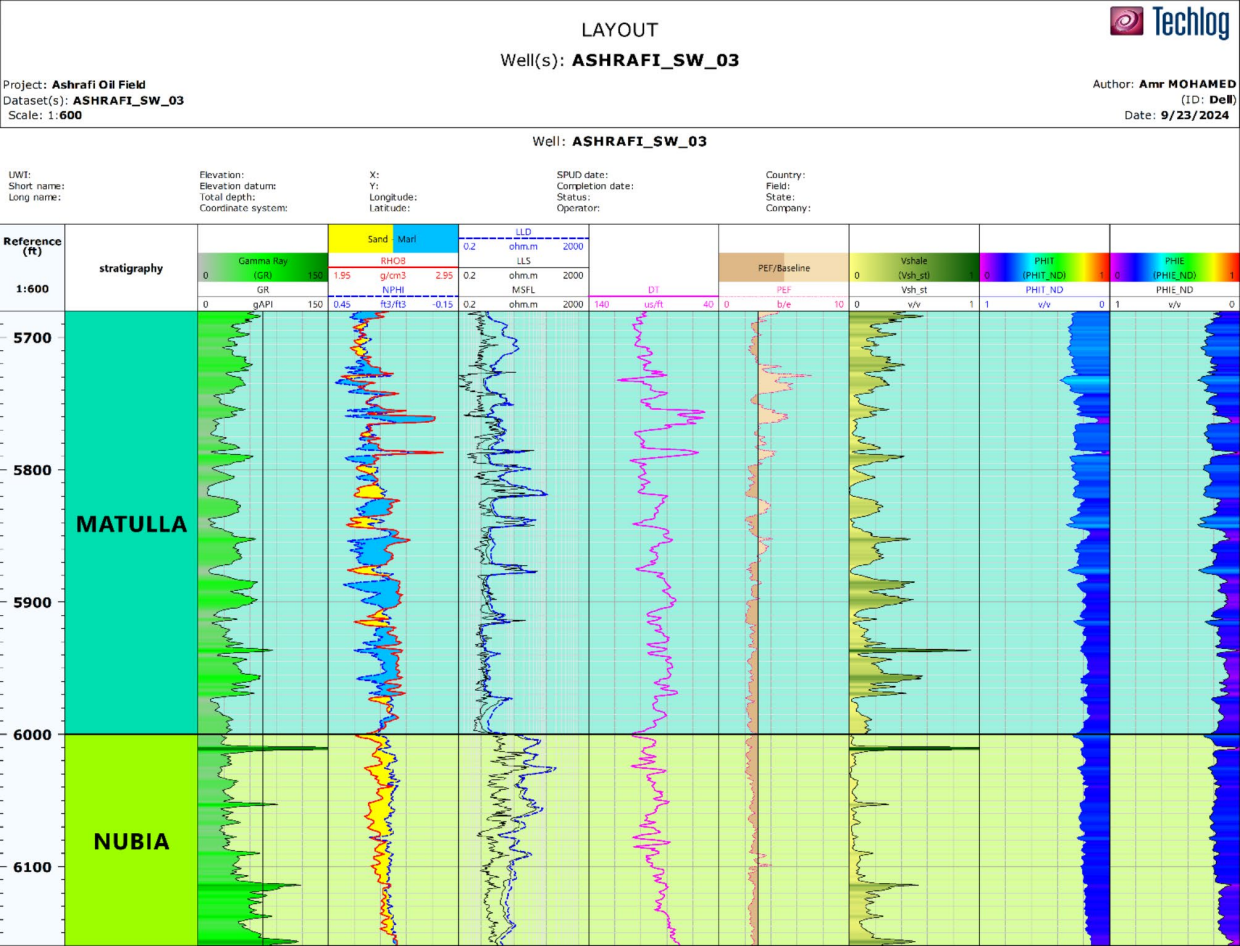
High-quality key well logs for Ashrafi_Sw_03 well.

The first step in the petrophysical analysis focused on reviewing the log displays and conducting a quality control process to verify the accuracy and reliability of the data. This careful examination of the well log data from key wells in the Ashrafi Oil Field was vital for identifying any inconsistencies or irregularities that could impact the subsequent analyses. By ensuring the precision and consistency of the log data, we established a strong foundation for deriving valuable petrophysical parameters. This process is crucial, as it ensures that the interpretations and conclusions drawn from the data are based on trustworthy measurements, thereby strengthening the overall analysis. Figure 3 presents high-quality key well logs for Ashrafi_Sw_03 well^26^.

### Zone of interest selection

Key zones within the formations were identified through an in-depth analysis of log signatures, which facilitated the delineation of distinct lithological units and their characteristics. Special attention was given to the Hammam Faraun Member, noted for its strong hydrocarbon potential due to its favorable sandy facies, good porosity, and high deep resistivity values indicating an excellent capacity for hydrocarbon storage and transmission^2,5,9,23,27,28^.

The Matulla and Nubia formations were also closely evaluated for their reservoir qualities. The Matulla Formation, with its sand-to-marl facies, was found to contain a significant volume of hydrocarbons, while the Nubia Formation, characterized by uniform, homogenous sandy facies, exhibited the highest hydrocarbon potential in the study area. This makes it a prime target for exploration activities. These thorough assessments provide critical insights into the reservoir potential of each formation, guiding future exploration and production efforts in the Ashrafi Oil Field (Fig. 3).

### Pickett plot

The Pickett plot is an essential tool in petrophysical analysis^29^, helping to determine key reservoir properties like the tortuosity factor (a), cementation exponent (m), saturation exponent (n), and the formation water resistivity (Rw). These parameters are critical for understanding how fluids, such as oil and water, move through the reservoir and for accurately calculating hydrocarbon saturation. By plotting resistivity versus porosity on a log-log scale, the Pickett plot provides a clear visual representation that makes it easier to identify fluid types and estimate water saturation. This method allows for a more precise evaluation of reservoir quality, which is crucial for making informed decisions about exploration and production^10,23^ .

**Fig. 4:**
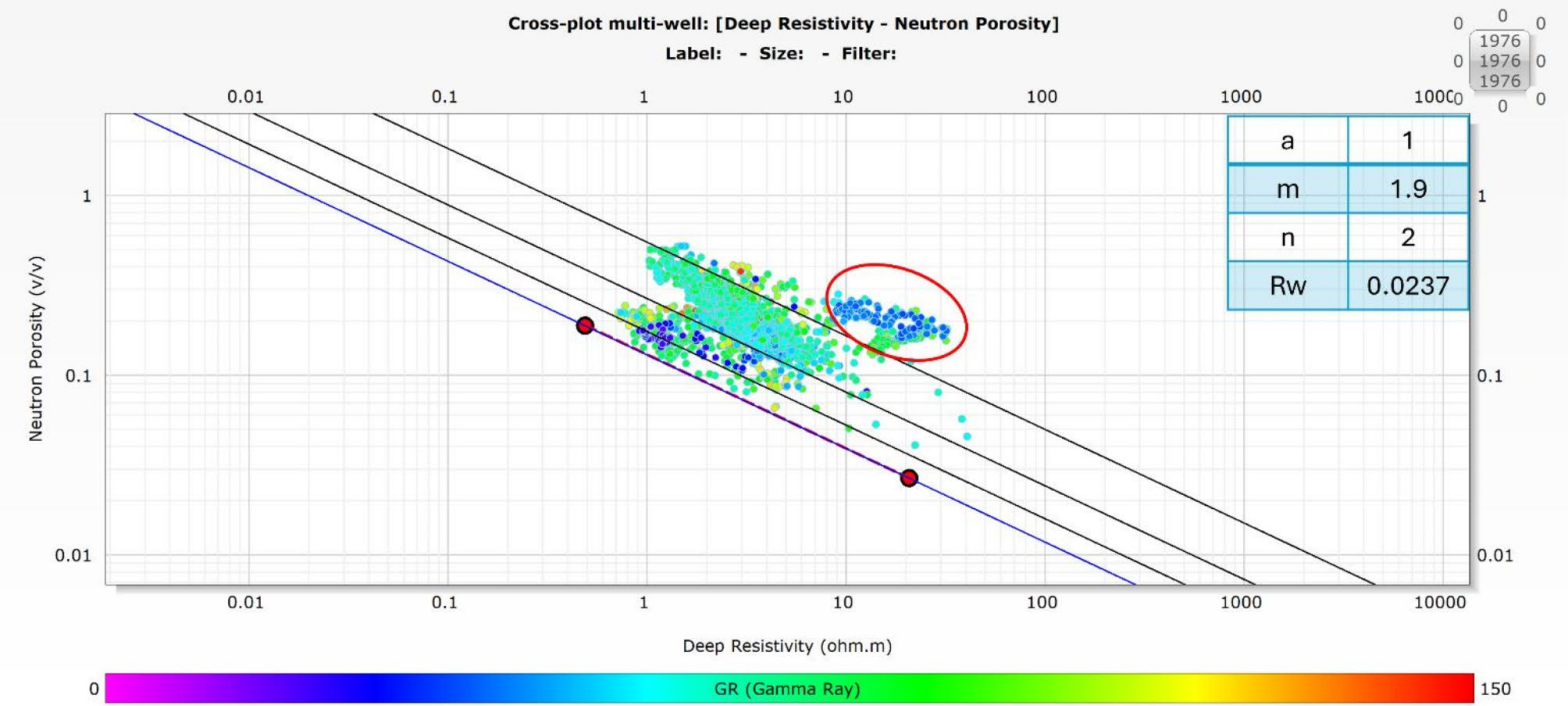
The Pickett plot for well ASHRAFI_404_B1X highlights the productive zone with a red circle, also displaying the extracted reservoir parameters.

In essence, the Pickett plot is a valuable step in understanding reservoir behavior and optimizing hydrocarbon extraction. Figure 4 presents the Pickett plot for well ASHRAFI_404_B1X.

### Matrix identification

The M-N crossplot was employed to accurately determine the lithology and matrix composition of the Hammam Faraun, Matulla, and Nubia formations in the Ashrafi Oil Field. This crossplot, a common petrophysical tool, plots the M (a function of density and neutron porosity) against the N (derived from sonic and neutron porosity) values, enabling the clear differentiation between various mineral constituents within the reservoir. By analyzing the position of data points on the M-N plot, the matrix composition of each formation can be identified, providing insights into the dominant minerals such as quartz, dolomite, calcite, or clay^2,10,23^. The Hammam Faraun, Matulla, and Nubia formations showed distinct trends on the crossplot, allowing for a more precise understanding of their lithological makeup.

The incorporation of RHOB (bulk density) and NPHI (neutron porosity index) plots is crucial for identifying the matrix in petrophysical analysis. These plots offer valuable insights into the lithological composition and attributes of subsurface formations, which are essential for thorough reservoir assessment.

When used together, RHOB and NHPI plot allow geoscientists to develop a detailed understanding of the subsurface matrix. By examining the relationships and trends presented in these plots, it becomes feasible to pinpoint potential reservoir rocks and assess their characteristics. This understanding ultimately aids in shaping exploration and production strategies. Accurately identifying matrix properties using RHOB and NHPI is vital for maximizing hydrocarbon recovery and achieving effective resource management in oil and gas exploration.

**Fig. 5:**
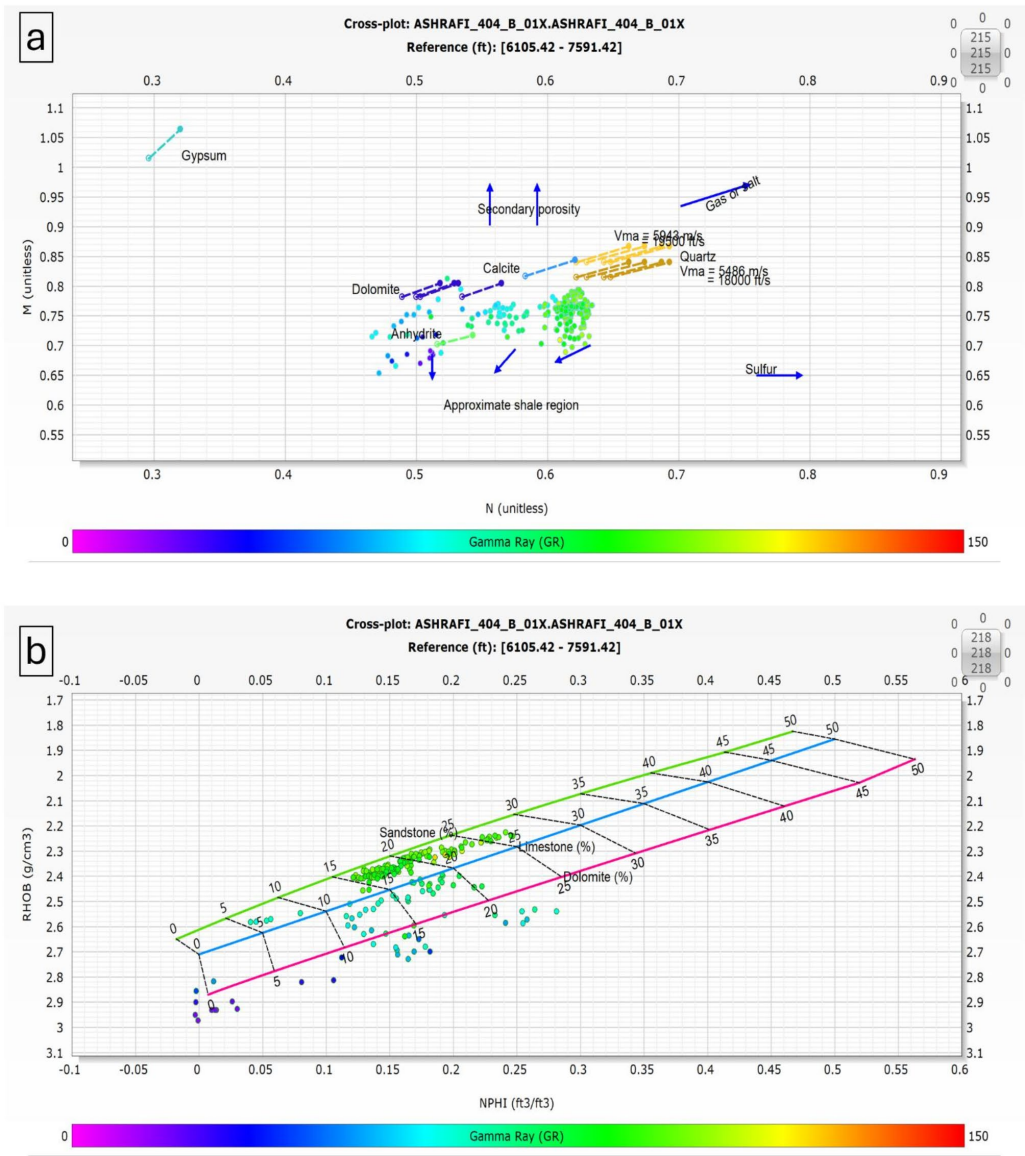
(a) M-N plot & (b) RHOB-NPHI Plot for Hammam Faraun Member.

**Fig. 6:**
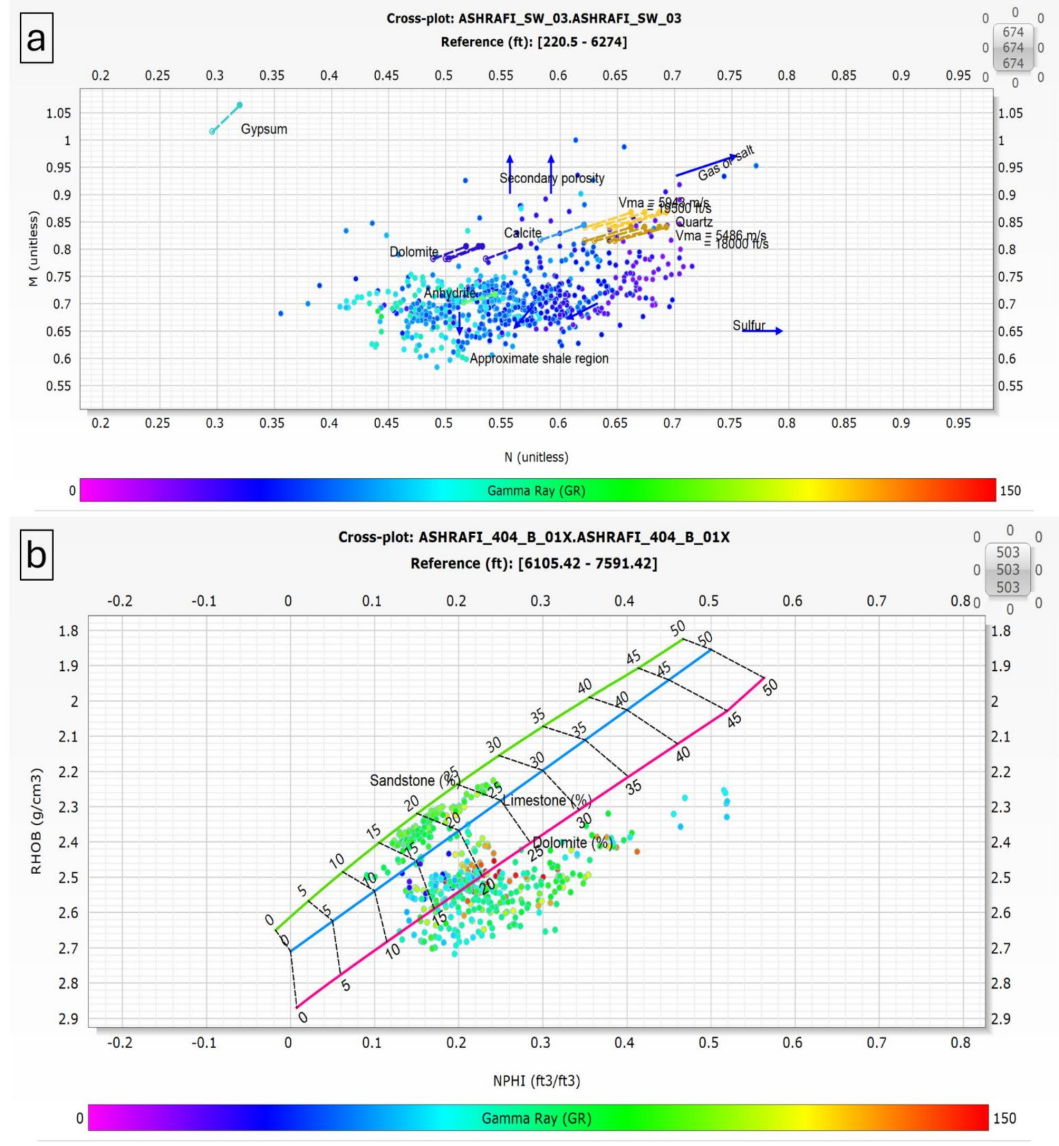
(a) M-N plot & (b) RHOB-NPHI Plot for Matulla Formation.

**Fig. 7:**
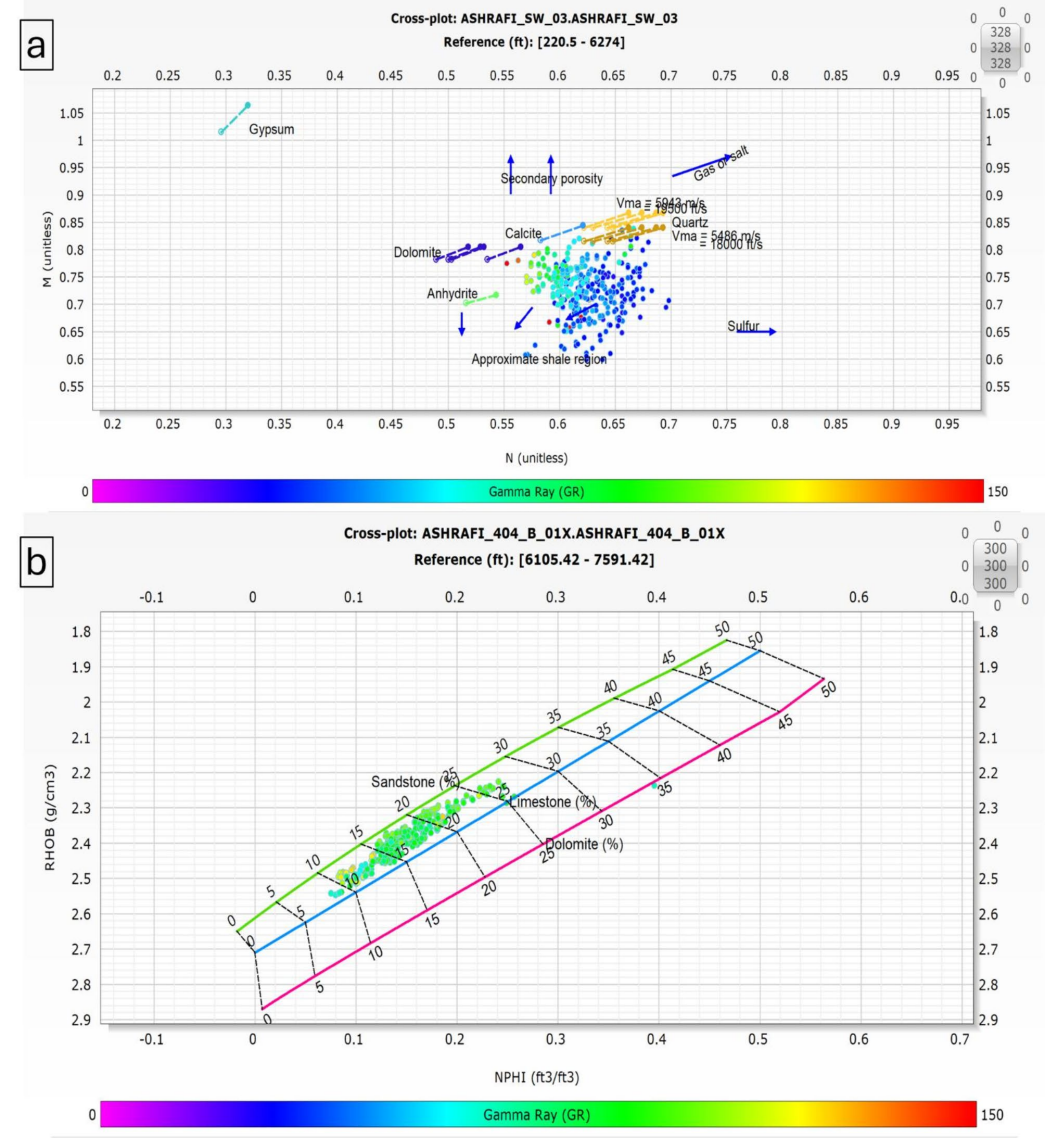
(a) M-N plot & (b) RHOB-NPHI Plot for Nubia Formation.

The Hammam Faraun Member is composed of feldspathic calcareous sand, with some shale and carbonate interbeds, grading to marl (Fig. 5a, b). The Matulla Formation comprises sandstone with interbedded marl and shale streaks (Fig. 6a, b). The Nubia Formation is primarily composed of pure sandstone (Fig. 7a, b). These two methods are crucial in reservoir characterization, as they enables accurate identification of the mineral framework, which in turn impacts porosity, permeability, and overall reservoir quality.

### Hydrocarbon indicators

**Fig. 8:**
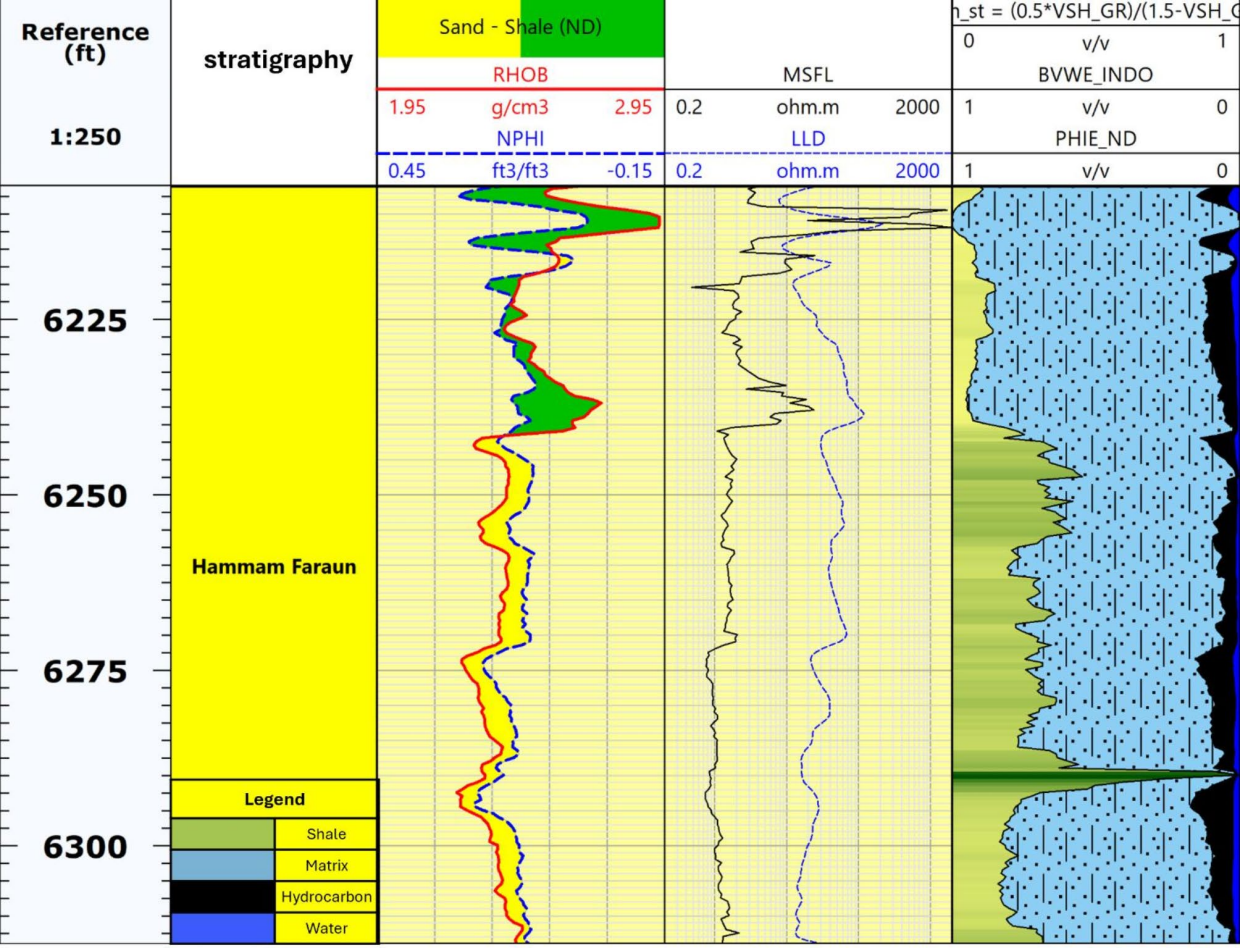
Hydrocarbon Indicator of Hammam Faraun Member in ASHRAFI_404_B1X Well.

**Fig. 9:**
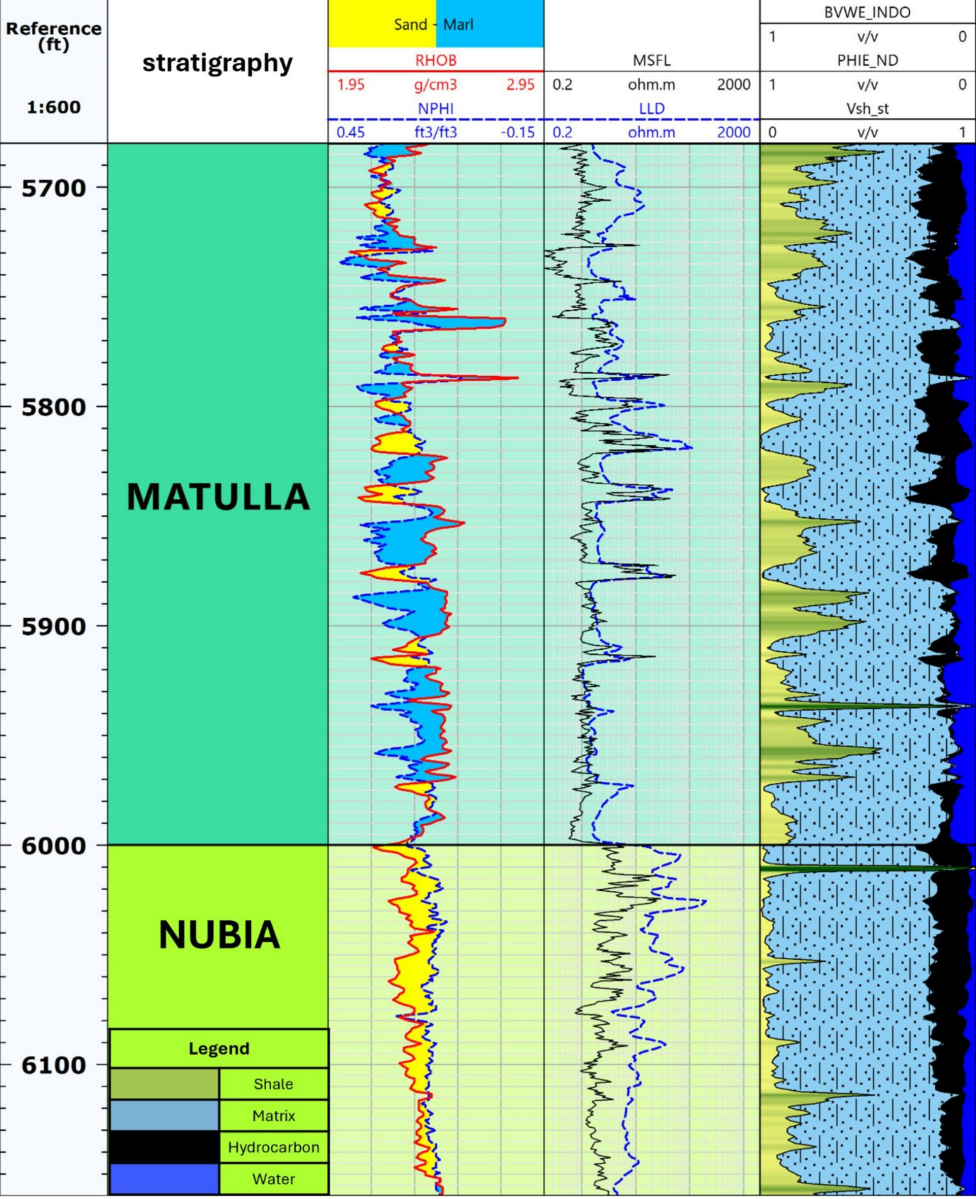
Hydrocarbon Indicator for Matulla & Nubia Formations in ASHRAFI_SW_03.

Hydrocarbon detection in the Hammam Faraun, Matulla, and Nubia formations of the Ashrafi Oil Field (Figs. 8 and 9) was based on key indicators such as the crossover between density and neutron logs and the differentiation of resistivity values^6,26^. A higher neutron log reading compared to the density log typically suggests the presence of oil, particularly if the separation is minimal; however, a larger separation often indicates gas, a process referred to as density-neutron crossover. Likewise, resistivity separation, where deep resistivity measurements are significantly greater than those of shallow resistivity, signals zones filled with hydrocarbons. These indicators are essential for differentiating hydrocarbon-rich areas from water-bearing formations, providing critical insights into the fluid types contained within the rock. By employing these techniques, we were able to more accurately identify the locations of hydrocarbons in the chosen zones, thereby enhancing our reservoir evaluation and informing exploration strategies. These methods not only facilitate hydrocarbon detection but also play a crucial role in optimizing production and ensuring effective long-term reservoir management^30^.

### Standard log data analysis

The standard logs (Gamma Ray, Neutron, Density & Resitivity) were used to calculate the fundamental petrophysical parameters: Volume of Shale, Effective Porosity, and Water Saturation, across the Hammam Faraun member, Matulla, and Nubia formations has provided a comprehensive understanding of the reservoir characteristics in the Ashrafi Oil Field^2,3^.

### Shale volume (vsh)

The Volume of Shale was calculated by analyzing Gamma Ray log data, a method known for its effectiveness in distinguishing between shaly and clean formations. By utilizing this approach, it becomes possible to accurately quantify the shale content within the reservoir. Understanding the Volume of Shale is essential, as it significantly impacts the overall quality of the reservoir and directly influences the potential for hydrocarbon accumulation. High shale volumes can indicate reduced reservoir quality, potentially leading to lower hydrocarbon productivity^2,3,6,23^. Conversely, accurately assessing the Volume of Shale allows for a more informed evaluation of the reservoir’s characteristics, helping to predict the presence of hydrocarbons and guide exploration efforts. This analysis serves as a fundamental step in the comprehensive petrophysical evaluation process, ultimately aiding in the effective management and development of hydrocarbon resources within the Ashrafi Oil Field. Shale Volume was estimate using the following equation^31^:

1 $$\:Vsh\:=\frac{{GR}_{log}-\:{GR}_{min}}{{GR}_{max}\:-\:{GR}_{min}}$$

In this context, Vsh refers to the volume of shale, while GRlog denotes the gamma ray measurement obtained from the logs. The values GRmax and GRmin represent the highest and lowest gamma ray readings, respectively.

### Total porosity (ϕT) and effective porosity (ϕeff)

Effective Porosity was assessed to quantify the pore spaces that are available for fluid flow within the rock matrix. This measurement is vital, as it directly affects the reservoir’s ability to store hydrocarbons and influences fluid movement. High Effective Porosity typically indicates a greater capacity for hydrocarbon retention, making it a key factor in evaluating reservoir performance. In order to calculate the effective porosity, the following equations were used:

The total porosity was calculated using the density and neutron logs, following the equation established by^31^:

2 $$\:{{\varnothing}}_{T}=\:\frac{{{\varnothing}}_{N}+\:{{\varnothing}}_{D}\:}{2}$$

In this equation, ΦT represents the total porosity, while ΦN indicates the porosity derived from the neutron log, and ΦD signifies the porosity obtained from the density log. The density porosity (ΦD) can be estimated based on the following equation^31^:

3 $$\:{{\varnothing}}_{T}=\:\frac{{{\varnothing}}_{N}+\:{{\varnothing}}_{D}\:}{2}$$

In this expression, ΦD denotes the porosity calculated from the density log. Additionally, ρm refers to the matrix density, ρb represents the rock’s bulk density, and ρfl indicates the density of the fluids occupying the pore spaces, which may include fresh or salt water and hydrocarbons.

The effective porosity was calculated using the formula outlined below^31,32^:

4 $$\:{{\varnothing}}_{eff}=\:{{\varnothing}}_{T}-\left(\:{V}_{sh}\text{*}\:{{\varnothing}}_{sh}\right)$$

In this formula, Φeff represents the effective porosity, while ΦT denotes the total porosity. Additionally, Vsh indicates the volume of shale, and Φsh refers to the porosity of the shale^2,3,10^.

### Water saturation (sw)

Water Saturation was calculated using the Indonesian equation, which estimates the percentage of pore volume occupied by water. This metric is important for assessing the viability of hydrocarbon extraction, as lower Water Saturation generally suggests a higher chance of oil or gas presence in the reservoir. The water saturation can be estimated based on the following formula:

5 $$\:\frac{1}{\sqrt{{R}_{t}}}=\left[\sqrt{\frac{{\phi\:}^{m}}{a{R}_{w}}}+\frac{{{V}_{cl}}^{\left(\frac{1-{V}_{cl}}{2}\right)}}{\sqrt{{R}_{cl}}}\right]{{S}_{w}}^{n}$$

In this equation, Sw signifies water saturation, Rt represents true resistivity, and Vcl denotes the volume of clay or shale. Additionally, ∅T indicates total porosity, ∅sh refers to shale porosity, a represents the tortuosity factor, Rw stands for the resistivity of formation water, and Rcl denotes the resistivity of the shale^9,12^.

### Bulk volume of Water saturation (BVW)

The Bulk Volume of Water Saturation (BVW) refers to the total volume of water contained within the pores of a rock formation. It is a crucial parameter in petrophysical analysis as it helps determine the amount of water present in relation to the overall volume of the rock, influencing the assessment of hydrocarbon reserves. This can be calculated using the equation provided below^33^:

6 $$BVW = SW{\text{*~}}\emptyset _{{eff}}$$

In this equation, BVW represents the bulk volume of water, Sw indicates water saturation, and ∅eff denotes effective porosity^6,10,25^.

### Well correlation

**Fig. 10:**
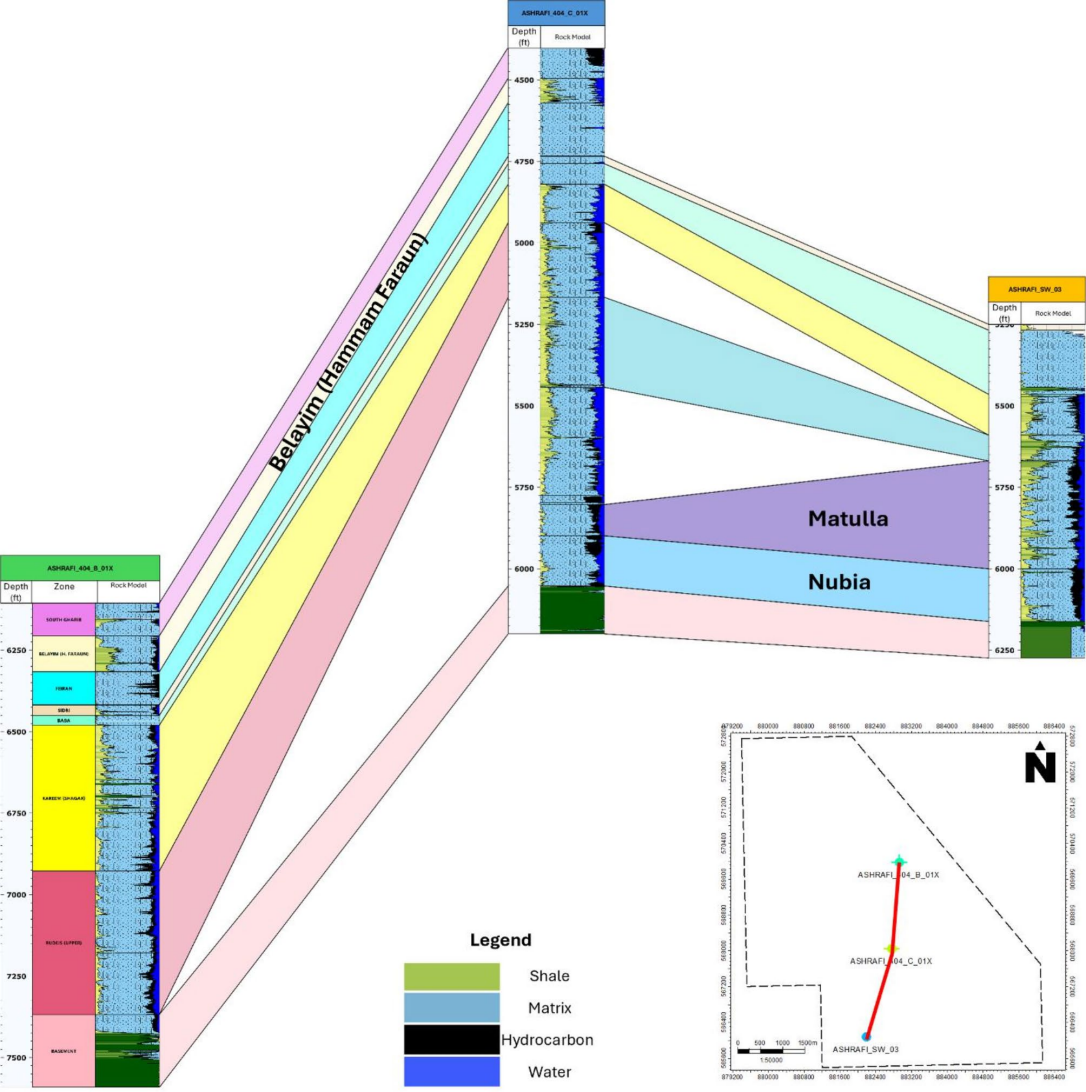
Well correlation showing the rock model of Hammam Faraun, Nubia and Matulla formations.

The well correlation was performed using wireline log data (Fig. 10), which facilitated the creation of a chart depicting thickness variations across different stratigraphic intervals within the study area. While the correlation extends from the Zeit Formation to the Basement, the primary focus is on the Belayim (Hammam Faraun Member), Matulla, and Nubia formations. The Belayim Formation is subdivided into four members: Hammam Faraun, Feiran, Sidri, and Baba. The Baba Member is predominantly composed of anhydrite with interbedded shale, while the Sidri Member mainly consists of anhydrite with some sand layers. The Feiran Member contains both anhydrite and salt, whereas the Hammam Faraun Member is primarily made up of feldspathic calcareous sand, with occasional shale and carbonate interbeds, transitioning to marl toward the northern area (Fig. 5). The thickness of the Hammam Faraun Member varies across the region, thinning towards the southwest. The Matulla Formation is characterized by sandstone with interbedded marl and shale layers (Fig. 6), while the Nubia Formation mainly consists of pure sandstone (Fig. 7). Thickness variations in both the Matulla and Nubia formations are observed across the area, largely due to faulting and unconformities, with a general thinning trend toward the northeast(Fig. 10).

### Lateral variation in parameters

The petrophysical analysis of the key zones: Hammam Faraun, Matulla, and Nubia, was performed using the Techlog geoscience platform. Data from neutron, density, resistivity, sonic, and gamma ray logs were employed to assess total and effective porosity, shale content, water saturation, hydrocarbon saturation, as well as net thickness across the target intervals.

Shale volume (Vsh) was calculated using Eq. (1), revealing variations across the different formations, Vsh maps were produced for each zone. Effective porosity, calculated using Eq. (4), demonstrates notable variability. Maps of porosity distribution across the study area highlight lateral differences. Water saturation was estimated using the Indonesian model (Eq. 5) to account for shale presence. Water saturation maps show lateral variation throughout the area, likely linked to facies changes. Net pay thickness was calculated using standard cut-off criteria.

**Fig. 11:**
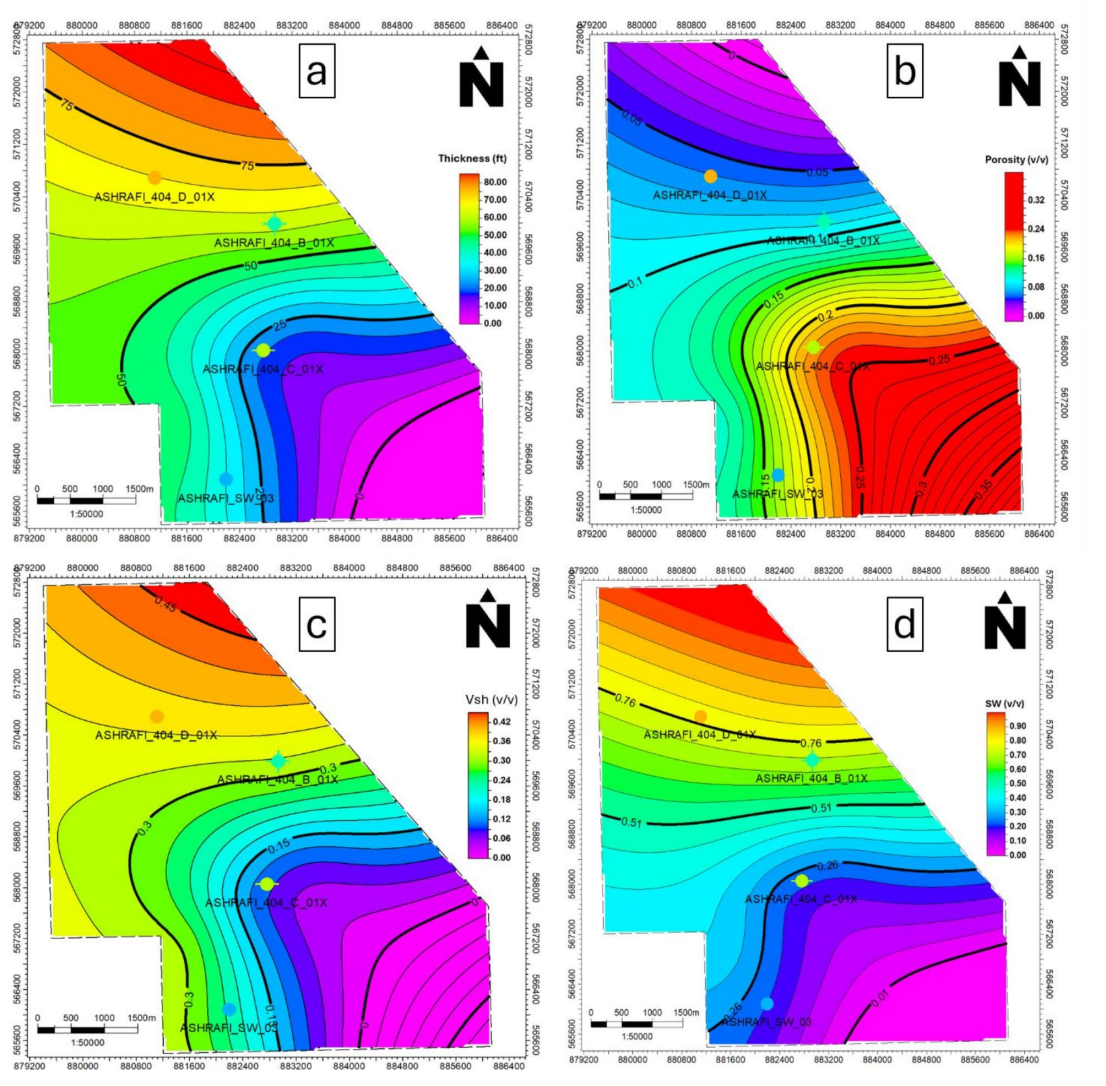
(a) Thickness map of the Hammam Faraun zone, (b) Porosity map of the Hammam Faraun zone, (c) Shale volume map of the Hammam Faraun zone and (d) Water saturation map of the Hammam Faraun zone.

In the Hammam Faraun zone, Vsh values range between 0.1 and 0.3, with lower values found toward the southeast (Fig. 11c), effective porosity varies from 0.09 to 0.22, with the highest values in the southwestern part (Fig. 11b), water saturation ranges from 0.23 to 0.67, with the lowest values in the southeast (Fig. 11d), net pay thickness ranges from 21 to 60 ft, with the thickest sands located in the northern region (Fig. 11a).

**Fig. 12:**
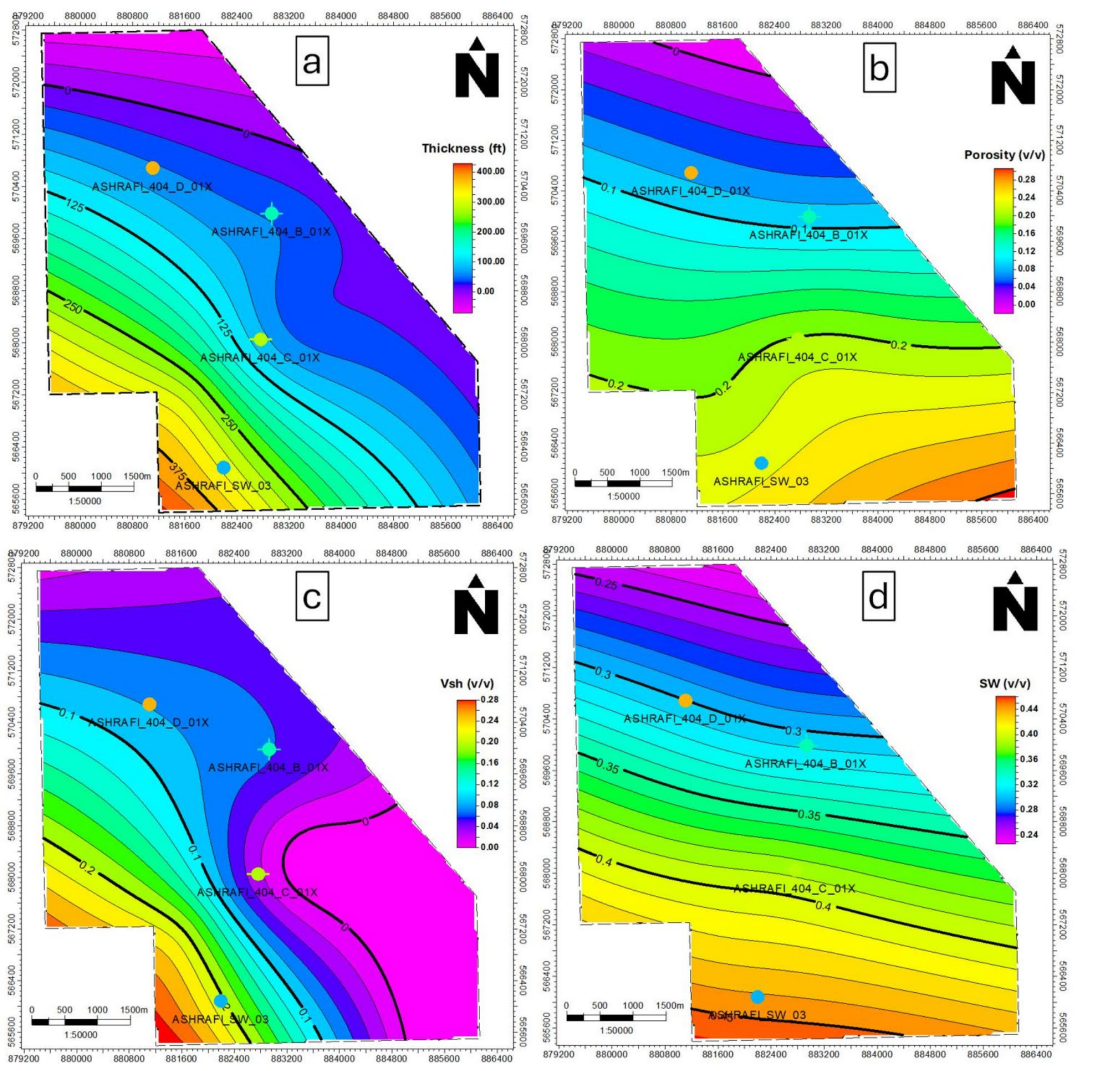
(a) Thickness map of the Matulla formation, (b) Porosity map of the Matulla formation, (c) Shale volume map of Matulla formation and (d) Water saturation map of the Matulla formation.

The Matulla zone exhibits a Vsh range from 0.02 to 0.18, with the eastern portion displaying the lowest shale content (Fig. 12c), effective porosity values between 0.1 and 0.2, peaking in the southeastern region (Fig. 12b), water saturation ranges from 0.31 to 0.41, with higher levels in the northern region (Fig. 12d), has a net pay thickness between 51 and 269 ft, with the largest thickness observed in the southeast (Fig. 12a).

**Fig. 13:**
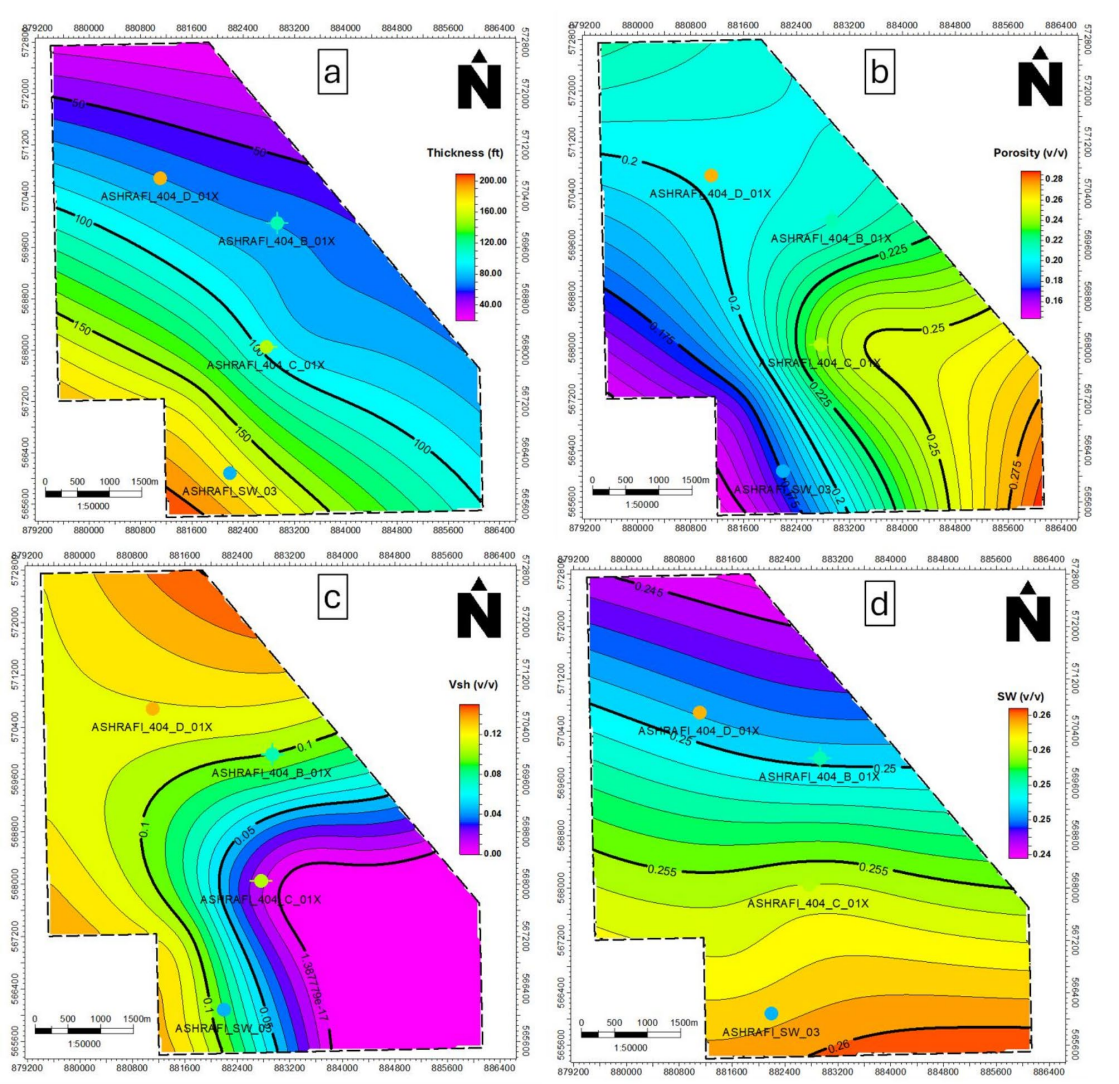
(a) Thickness map of the Nubia formation, (b) Porosity map of the Nubia formation, (c) Shale volume map of Nubia formation and (d) Water saturation map of the Nubia formation.

The Nubia zone shows consistently low shale volume, ranging from 0.013 to 0.09 (Fig. 13c), Nubia, ranging from 0.18 to 0.23, has its best porosity in the southeast (Fig. 13b), The Nubia zone has a uniform water saturation of approximately 0.24 across the area (Fig. 13d), net pay ranges from 72 to 155 ft, with maximum thickness in the southeastern part of the study area (Fig. 13a).

The variability in Hammam Faraun and Matulla is likely due to facies heterogeneity, while Nubia appears more uniform, indicating homogeneity in its facies.

### Litho-saturation model

The rock model provides a detailed representation of the bulk rock volume composition, integrating three critical petrophysical parameters: shale volume (Vsh), effective porosity (ϕ_eff), and bulk volume of water (BVW). By analyzing the model, it is possible to estimate the matrix percentage, shale volume, and hydrocarbon saturation within the formation^6,10,25^. Serving as the culmination of petrophysical analysis, the rock model is crucial for interpreting reservoir quality, highlighting the zones with the highest potential for hydrocarbon storage and production.

The model was developed for three key formations: the Hammam Faraun Member of the Belayim Formation, the Matulla Formation, and the Nubia Formation, all of which displayed favorable petrophysical characteristics, identifying them as potential hydrocarbon-bearing zones. According to the litho-saturation model (Figs. 8, 9 and 10), these formations exhibit promising conditions for hydrocarbon accumulation, with strong porosity and saturation values. The Hammam Faraun Member, in particular, shows an ideal balance between reservoir quality and fluid saturation, while the Matulla and Nubia formations demonstrate good hydrocarbon potential across the study area. This model, built upon wireline log data and petrophysical analysis, serves as a key tool in understanding subsurface characteristics, enhancing hydrocarbon resource estimates, and guiding exploration and development decisions.

### Seismic interpretation

The seismic data analysis for the Ashrafi Oil Field was carried out in four major steps: (1) detection of formation tops and seismic-to-well tie, (2) lateral delineation of horizons and fault detection, (3) and closure of loops and creation of geological cross-sections, and (4) defining the major trend of the key features^3,28^. The main objective of this analysis was to identify oil and gas accumulations, track their lateral extent.

**Fig. 14:**
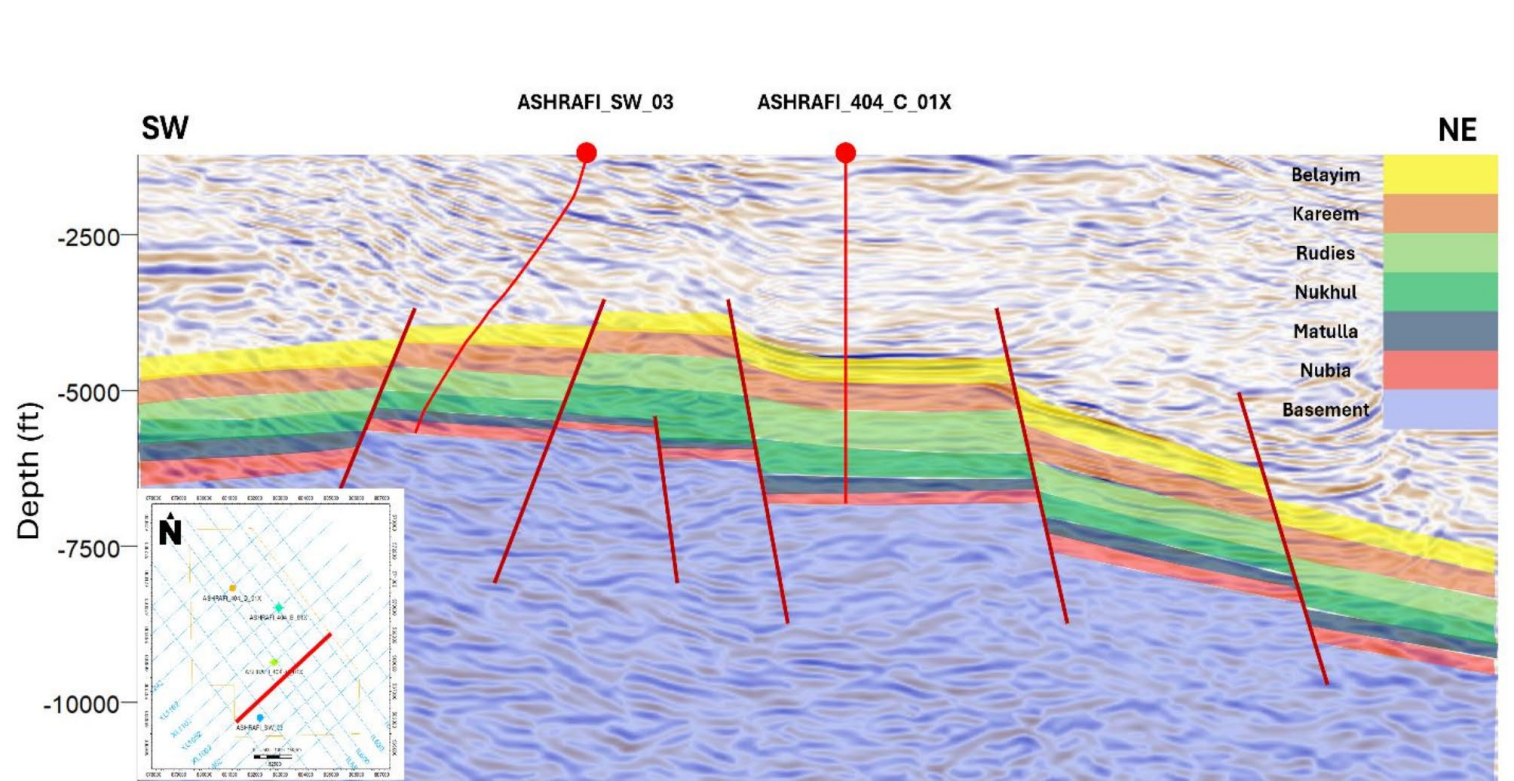
Interpreted seismic section from the southern part of the area, highlighting the present structural features.

Formation tops were identified and tracked laterally in the seismic sections, with particular focus on the Belayim (Hammam Faraun Member), Matulla, and Nubia formations. Faults were also interpreted and mapped across the seismic sections. The resulting interpretation revealed a series of normal faulted structures, including horsts, half-grabens, and step faults (Fig. 14). These faults extend throughout the area, predominantly trending northeast-southwest (clysmic trend), with minor northwest-southeast faults perpendicular to the major faults, forming a complex fault network. These major and minor faults influence the reflectors of interest, namely the Hammam Faraun, Matulla, and Nubia formations.

Further analysis indicates that the basement rises toward the southwest, causing a thinning of the sediment column in that direction. Conversely, the sediment column thickens toward the northeast, where a grater thickness of sediment is observed. This observation is consistent with the well correlation and petrophysical analysis. The tectonic configuration of the area, located in the southern part of the Gulf, serves as a useful analog for the Red Sea, which encounters similar geological challenges, including salt presence and limited sediment thickness.

The interpretation reveals a promising petroleum system with three reservoirs, characterized by complex fault traps (Fig. 14) sealed by overlying salts. These faults also serve as migration pathways for hydrocarbons, enhancing the reservoir’s potential.

### Petroleum system

The petroleum system of the Ashrafi Oil Field is defined by a complex relationship between the source, reservoir, seal, and migration paths. The primary source rocks for hydrocarbons in this area are organic-rich shales within the Hammam Faraun and Matulla formations, which generate hydrocarbons through thermal maturation. These hydrocarbons migrate through fault systems that cut across the field, with the Hammam Faraun, Matulla, and Nubia formations serving as the key reservoirs. The reservoir quality varies due to lithological heterogeneity, with significant variations in porosity and permeability. The salt layers, along with fault-related traps such as horsts, half-grabens, and step faults, function as effective seals, ensuring the trapping of hydrocarbons within the reservoir. Tectonic activity, particularly faulting, plays a crucial role in creating migration pathways for hydrocarbons, allowing them to accumulate in structurally favorable traps (Fig. 14). These combined elements: source, reservoir, seal, and migration pathways establish a highly promising petroleum system in the Ashrafi Oil Field, enhancing its hydrocarbon potential.

## Discussion

In this study, a comprehensive petrophysical and reservoir characterization analysis was conducted for the Hammam Faraun, Matulla, and Nubia formations within the Ashrafi Oil Field. Our findings not only align with but also build upon previous regional studies, offering new insights into the lithological composition, hydrocarbon potential, and petrophysical properties of these formations. The integration of M-N crossplots, RHOB, and NPHI plots allowed for a more refined understanding of the lithology, facies distribution, and hydrocarbon saturation across the formations.

One of the key contributions of this study is the detailed identification of mineral composition and lithological variations in the Hammam Faraun, Matulla, and Nubia formations. The Hammam Faraun Member was found to primarily consist of feldspathic calcareous sand with interbedded shale and carbonate layers, while the Matulla Formation predominantly comprises sandstone, with notable interbeds of marl and shale. The Nubia Formation, on the other hand, is composed almost entirely of pure sandstone. These lithological distinctions, particularly within the Hammam Faraun Member, provide a more precise characterization than prior studies such as^13,34^ ,which offered broader descriptions. This more granular lithological interpretation is crucial for future reservoir modeling as it influences porosity, permeability, and overall reservoir quality.

Additionally, our analysis of lateral variability in petrophysical parameters such as shale volume (Vsh), effective porosity, water saturation, and net pay thickness highlights significant spatial heterogeneity within the Ashrafi Oil Field. Notably, the Hammam Faraun formation displays significant lateral variations in porosity and water saturation, with higher porosity values in the southwest and lower saturation in the southeast. This finding is particularly important because earlier studies (e.g^35,36^). , did not provide such detailed lateral distribution within the Hammam Faraun Member. The southeastern region, with its lower water saturation, is identified as a prime target for hydrocarbon extraction, offering a new perspective not strongly emphasized in previous research.

In the Matulla Formation, we observed variations in shale volume distribution, with the eastern section exhibiting lower shale content, contributing to higher porosity and potentially greater hydrocarbon saturation in that region. Previous works, such as^37^, recognized the Matulla Formation as a major target for oil, but did not focus on the lateral distribution of shale content and porosity as comprehensively as our study. The heterogeneity within the Matulla Formation, especially in terms of reservoir quality, has significant implications for oil extraction.

The Nubia Formation, in contrast, exhibited more uniform petrophysical properties, with consistent low shale volume and moderate porosity throughout the field. This homogeneity, highlighted in previous studies by^38^, suggests a more stable reservoir compared to the more heterogeneous Hammam Faraun and Matulla formations. The consistent low water saturation further enhances its appeal for hydrocarbon extraction.

A novel aspect of our study is the development of a litho-saturation model integrating shale volume (Vsh), effective porosity (ϕ_eff), and bulk volume of water (BVW). This new model reveals promising hydrocarbon saturation levels, particularly in the Hammam Faraun and Matulla formations, where regions with higher porosity and lower water saturation show greater hydrocarbon potential. This model provides a more accurate prediction of hydrocarbon saturation compared to earlier models from^34,35^, enabling better resource estimation.

Furthermore, our use of well correlation has uncovered detailed stratigraphic thickness variations across the Ashrafi Oil Field. While earlier studies observed thinning formations toward the southwest and northeast, our research emphasizes the role of faulting and unconformities in these thickness variations, especially in the Hammam Faraun and Nubia formations. Faulting, previously overlooked in studies like^39^, may significantly affect reservoir management and the distribution of hydrocarbon accumulations.

Finally, our seismic data analysis revealed a complex network of normal faults that control hydrocarbon migration and accumulation, in line with studies such as^34,35^. However, our more detailed fault mapping, especially concerning minor northwest-southeast faults, enhances the understanding of their impact on the Hammam Faraun, Matulla, and Nubia formations. Our findings on sediment thickness variations, with a rising basement to the southwest and thickening sediment to the northeast, align with previous petrophysical analyses and well correlations. These structural features, combined with salt-sealed traps, are crucial in defining the petroleum system within the Ashrafi Oil Field.

Finally, our study provides several new findings that enhance the understanding of reservoir properties in the Ashrafi Oil Field. These include more detailed lithological identification, a comprehensive analysis of lateral variations in petrophysical parameters, and a novel litho-saturation model that offers new insights into hydrocarbon potential. These results not only corroborate previous studies but also offer a deeper and more refined perspective on the reservoir quality, which can significantly influence future exploration, production strategies, and hydrocarbon recovery in the Ashrafi Oil Field.

## Conclusion

This study delivers an extensive evaluation of the Hammam Faraun, Matulla, and Nubia formations within the Ashrafi Oil Field, concentrating on their composition, crucial petrophysical parameters, and structural settings essential for hydrocarbon exploration and production.

The Hammam Faraun Formation is recognized for its advantageous sandy facies, with effective porosity values ranging from 0.15 to 0.25. This formation exhibits substantial hydrocarbon storage potential, especially in the northern region, where the net pay thickness can reach up to 60 ft. Water saturation levels, which fluctuate between 0.23 and 0.67, indicate a promising capacity for hydrocarbon extraction, making this formation a primary target for further exploration.

Conversely, the Matulla Formation has a more intricate lithological composition, with sand-to-marl facies contributing to effective porosity values between 0.10 and 0.20. Its water saturation levels range from 0.31 to 0.41, and it displays significant net pay thickness varying from 51 to 269 ft. This variability highlights the Matulla Formation’s considerable hydrocarbon volumes and its viability as a production reservoir.

The Nubia Formation, characterized by its uniform and homogenous sandy facies, shows effective porosity values around 0.18 and a water saturation of approximately 0.24. With net pay thicknesses ranging from 72 to 155 ft, the Nubia Formation emerges as a strong candidate for hydrocarbon exploration due to its favorable reservoir attributes.

The three formations of interest are influenced by a series of normal faults throughout the area, with a major northeast-southwest trend (clysmic trend) and minor northwest-southeast faults perpendicular to the major trend. These faults create a variety of faulted traps, including horsts, half-grabens, and step faults, which are capable of trapping significant amounts of hydrocarbons.

In summary, the petrophysical analysis plays a crucial role in enhancing the understanding of reservoir characteristics in the Ashrafi Oil Field, specifically within the Hammam Faraun, Matulla, and Nubia formations. By integrating key parameters such as shale volume, effective porosity, and water saturation, this research provides valuable insights into reservoir quality and fluid distribution. Recent well log data have identified previously overlooked fault lines and fluid flow pathways, offering a more comprehensive view of the reservoir’s heterogeneity and suggesting the presence of additional hydrocarbon reserves. These findings are essential for optimizing extraction techniques and improving recovery efforts, leading to better resource management in the Gulf of Suez. Moreover, the enhanced petrophysical insights from this study have broader applications for exploration in similar geological settings, including the Red Sea region, providing valuable guidance for future exploration and development strategies.

## Data Availability

The corresponding author has to be contacted in case of any queries or requirements of data.
